# Engineering the stereoisomeric structure of seed oil to mimic human milk fat

**DOI:** 10.1073/pnas.1907915116

**Published:** 2019-09-30

**Authors:** Harrie van Erp, Fiona M. Bryant, Jose Martin-Moreno, Louise V. Michaelson, Govindprasad Bhutada, Peter J. Eastmond

**Affiliations:** ^a^Plant Science Department, Rothamsted Research, Harpenden, AL5 2JQ Hertfordshire, United Kingdom

**Keywords:** metabolic engineering, oilseeds, structured triacylglycerols, human milk fat

## Abstract

Human milk fat substitute (HMFS) is a class of structured lipid that is widely used as an ingredient in infant formulas. Like human milk fat, HMFS is characterized by enrichment of palmitoyl (C16:0) groups specifically at the middle (sn-2 or β) position on the glycerol backbone, and there is evidence that triacylglycerol (TAG) with this unusual stereoisomeric structure provides nutritional benefits. HMFS is currently made by in vitro enzyme-based catalysis because there is no appropriate biological alternative to human milk fat. Most of the fat currently used in infant formulas is obtained from plants, which exclude C16:0 from the middle position. In this study, we have modified the metabolic pathway for TAG biosynthesis in the model oilseed *Arabidopsis thaliana* to increase the percentage of C16:0 at the middle (vs. outer) positions by more than 20-fold (i.e., from ∼3% in wild type to >70% in our final iteration). This level of C16:0 enrichment is comparable to human milk fat. We achieved this by relocating the C16:0-specific chloroplast isoform of the enzyme lysophosphatidic acid acyltransferase (LPAT) to the endoplasmic reticulum so that it functions within the cytosolic glycerolipid biosynthetic pathway to esterify C16:0 to the middle position. We then suppressed endogenous LPAT activity to relieve competition and knocked out phosphatidylcholine:diacylglycerol cholinephosphotransferase activity to promote the flux of newly made diacylglycerol directly into TAG. Applying this technology to oilseed crops might provide a source of HMFS for infant formula.

## Significance

In human milk fat, saturated fatty acids are esterified to the middle position on the glycerol backbone giving the triacylglycerol molecules an unusual stereochemistry that assists nutrient absorption in the infant gut. However, the fat used in most infant formulas is derived from plants, which esterify saturated fatty acids to the outer positions. Here, we have engineered the metabolism of an oilseed plant so that it accumulates triacylglycerol with more than 70% of the saturated fatty acid palmitate in the middle position, thereby mimicking human milk fat stereoisomeric structure. Applying this technology to oilseed crops (or oleaginous microorganisms) might provide a source of human milk fat substitute for infant nutrition.

Infant formula is a manufactured food designed to substitute for human breast milk. Around half the calories in human milk are provided by fat (triacylglycerol; TAG) and in infant formula this fat is mainly sourced from plants (1). Although blended vegetable fats can replicate the fatty acyl composition of human milk fat (HMF), which mainly comprises palmitate (C16:0) and oleate (C18:1), the arrangement of acyl groups esterified to the glycerol backbone (i.e., the stereoisomeric structure) is profoundly different (2, 3). In vegetable fats, saturated long-chain fatty acyl groups such as C16:0 occupy the outer stereospecific numbering (sn) positions (sn-1/3) and are virtually excluded from the middle (sn-2 or β) position (4, 5); whereas in HMF, more than 70% of the C16:0 is present at the sn-2 position, with unsaturated fatty acyl groups (mainly C18:1) occupying the outer sn-1/3 positions (2, 3).

Multiple clinical trials on infants have suggested that the unusual stereoisomeric structure of HMF is important for nutrient absorption in the neonatal gut (1, 3, 6). The proposed mechanism is as follows. During the intestinal phase of digestion, lipases attack ingested fat at the sn-1/3 positions yielding 2-monoacylglycerols, which are easily absorbed (1, 3, 6). When unsaturated fatty acids are released from sn-1/3 positions, they are also absorbed easily, but release of long-chain saturated fatty acids such as C16:0 presents a problem. Their melting point is higher than body temperature and, at intestinal pH, they are prone to form hydrated fatty acid soaps with minerals such as calcium and magnesium (1, 3, 6). The arrangement of C16:0 at the sn-1/3 positions of vegetable fats thus means that they are more poorly absorbed than HMF (1, 3). There is evidence that the formation of C16:0 soaps also reduces calcium absorption, thus impairing early bone development, and accumulation of these soaps in the intestine also disrupts transit, causing infants discomfort (1, 3, 6).

To mimic the stereoisomeric structure of HMF, several companies have developed HMF substitutes (HMFS) (1). HMFS are made by enzyme-catalyzed acidolysis (or alcoholysis and esterification) using tripalmitin, unsaturated free fatty acids (mainly C18:1) together with an immobilized recombinant sn-1/3-regioselective lipase (1). The price of HMFS is substantially higher than that of conventional vegetable fat blends, primarily reflecting the added cost of enzyme-based catalysis, which also generates organic solvent waste (7). Different grades of HMFS are also available, providing a complete fat phase with between ∼40 and ∼70% of C16:0 at the sn-2 position. True HMF mimetics (with >70% of C16:0 at sn-2) are most expensive to produce because they require a 2-step catalytic process and a pure tripalmitin feedstock derived from palm oil by special fractionation procedures and chemical randomization (1, 7). The tension between price and quality is one factor that has likely restricted the use of HMFS and despite mounting clinical evidence that this ingredient is beneficial (1, 3, 6), it is currently only found in around 10% of infant formula, particularly premium products formulated and marketed for ease-of-digestion. Even in these products, there remains a substantial gap in C16:0 enrichment at the sn-2 position versus HMF (1).

The aim of this study was to explore whether the stereoisomeric structure of vegetable fat can be altered by iterative metabolic engineering, so that it mimics HMF. To our knowledge, no land plant (Embryophyta) produces TAG enriched in C16:0 at the sn-2 (verses sn-1/3 positions) and C16:0 is largely excluded from this position in virtually all cases (4, 5, 8). Even in palm oil that contains ∼48% C16:0 in total, only 9% of this occupies the sn-2 position (5). Here, we describe a method for modifying TAG biosynthesis, in the model oilseed *Arabidopsis thaliana*, that results in a stereoisomeric redistribution of acyl groups such that the amount of C16:0 at the sn-2 position increases more than 20-fold to over 70% of the total; a level of enrichment that is comparable to HMF. Applying this technology to oilseed crops might provide a source of HMFS for infant formula.

## Results and Discussion

### LPAT1 Can Be Redirected to the ER by Removing Its Chloroplast Targeting Signal.

**Fig. 1.**
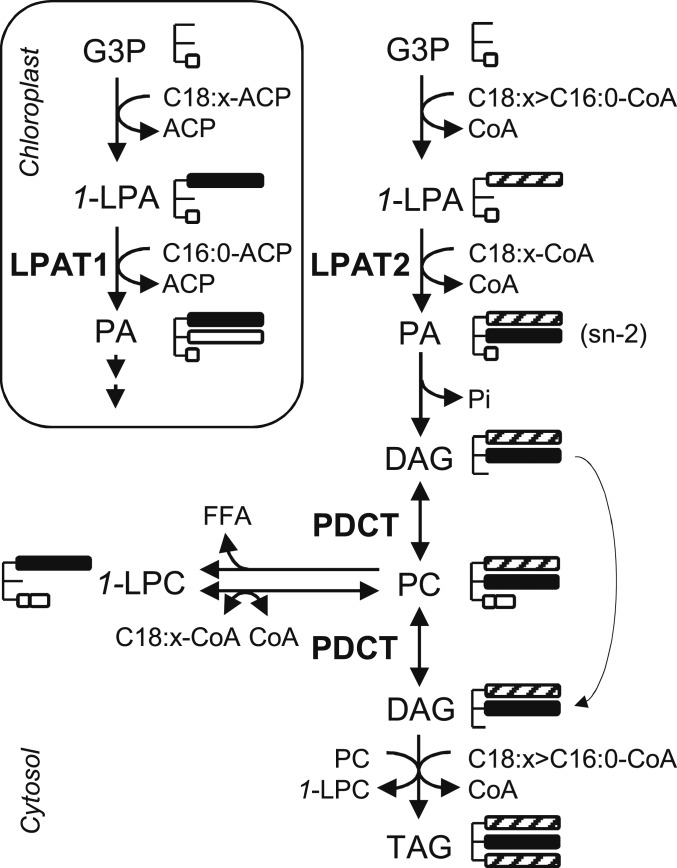
A simplified diagram illustrating the cytosolic and chloroplastic pathways for de novo glycerolipid biosynthesis in Arabidopsis. Three modifications enabled palmitoyl (C16:0) groups (white bars) to be incorporated into the sn-2 (or β) position of TAG in developing seeds. (1) Retargeting of LPAT1 to the ER, (2) knock down of LPAT2 and (3) knock out of PDCT. C18:x, long-chain mono-, or polyunsaturated fatty acyl groups (black bars); C16:0 and C18:x groups (hatched bars); CoA, CoenzymeA; ACP, acyl carrier protein; G3P, glycerol-3-phosphate; 1-LPA, sn-1 lysophosphatidic acid; PA, phosphatidic acid, DAG, diacylglycerol, TAG, triacylglycerol; PC, phosphatidylcholine; 1-LPC, sn-1 lysophosphatidylcholine; FFA, free fatty acid; LPAT, 1-LPA acyltransferase; PDCT, PC:DAG cholinephosphotransferase.

**Fig. 2.**
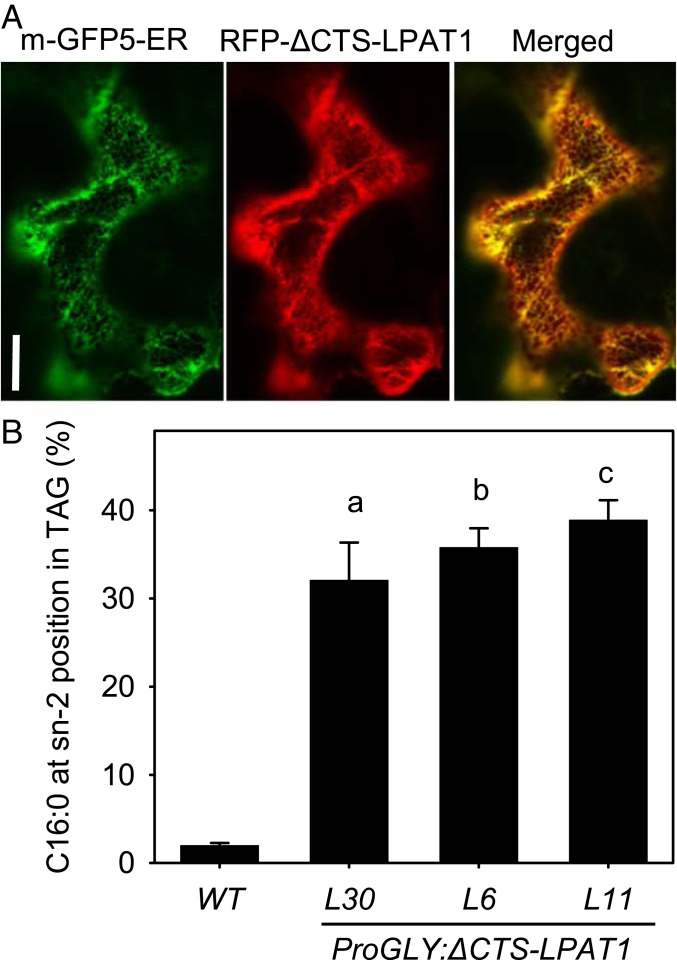
Chloroplast LPAT1 can be retargeted to the cytosolic glycerolipid biosynthetic pathway to incorporate C16:0 into the sn-2 position of TAG. (*A*) Laser scanning confocal microscopy image of a *N. benthamiana* epidermal cell transiently expressing RFP-∆CTS-LPAT1 and m-GFP5-ER marker. (Scale bar, 20 µm.) (*B*) Effect of seed-specific *∆CTS-LPAT1* expression in *Arabidopsis* on the percentage of C16:0 esterified to the *sn-2* position of TAG, verses sn-1+3. WT, wild type; L30, L6, and L11, 3 independent homozygous *ProGLY:∆CTS-LPAT1* lines. Values are the mean ± SE of measurements made on separate seed batches from 3 plants of each genotype (*n* = 3). a, b and c denote values significantly (*P* < 0.05) different from WT (ANOVA + Tukey HSD test).

In plant cells, triacylglycerol (TAG) is formed by a cytosolic glycerolipid biosynthetic pathway situated on the endoplasmic reticulum (ER) and the enzyme responsible for acylation of the sn-2 position is lysophosphatidic acid acyltransferase (LPAT) (9) (Fig. 1). ER-resident isoforms of LPAT commonly discriminate against C16:0-CoenzymeA (CoA) as a substrate, and this may be why C16:0 is excluded from the sn-2 position (9, 10). To overcome this limitation, we decided to express an LPAT with specificity for C16:0-CoA (Fig. 1). Several candidate transgenes have been described from cyanobacteria (11), mammals (12), and algae (13, 14). However, plants already possess an LPAT with the appropriate selectivity that resides in the chloroplast (15, 16) (Fig. 1). This LPAT uses a C16:0-acyl carrier protein (ACP) substrate but will also accept C16:0-CoA in vitro (17, 18). We therefore decided to test whether chloroplast LPAT could be relocated to the ER (Fig. 1). Chloroplast LPAT is an integral membrane protein that is nuclear encoded and contains an N-terminal chloroplast targeting signal (CTS) (19). CTS deletion has previously been used to alter protein localization (20). Using transient expression in *Nicotiana benthamiana* leaves, we found that when 101 amino acid residues containing the CTS are deleted from *Brassica napus* LPAT1 (17) (*SI Appendix*, Fig. S1) and replaced with a red fluorescent protein (RFP) marker, the RFP-ΔCTS-LPAT1 fusion protein localizes to the ER (Fig. 2*A*).

### ∆CTS-LPAT1 Expression Drives C16:0 Incorporation into the sn-2 Position of TAG.

Truncated versions of LPAT1 that lack the CTS are known to be active when expressed in *Escherichia coli* (18, 19). To determine whether ∆CTS-LPAT1 functions in plants and can enable C16:0 to be incorporated into the sn-2 position of TAG, we expressed this truncated protein under the control of the seed-specific soybean glycinin-1 promoter (*ProGLY*) in the model oilseed *Arabidopsis* (21). We selected more than 40 primary transformants (T1) using a DsRed fluorescent marker system (21) and analyzed the total fatty acyl composition of T2 seed batches. We found that several lines exhibited an increase in total C16:0 content, which suggested that the transgene was promoting C16:0 incorporation into TAG (*SI Appendix*, Table S1). We selected 3 independent single copy T2 lines (L30, L6, and L11) with high C16:0 content and obtained homozygous T3 seed. When we purified TAG from these homozygous seed batches and determined its stereochemistry using lipase digestion (22), we found that the percentage of C16:0 at the sn-2 position (vs. sn-1+3), had increased more than 16-fold, from only ∼2% in wild type to values ranging between ∼32 and ∼39% in the 3 independent *ProGLY:∆CTS-LPAT1* lines (Fig. 2*B* and *SI Appendix*, Table S2). *∆CTS-LPAT1* expression was therefore sufficient to allow incorporation of C16:0 into the sn-2 position of TAG, but not to achieve positive enrichment at this position verses the sn-1/3 positions, which can already incorporate a low proportion of C16:0 (9) (Fig. 1).

### Disruption of LPAT2 Enhances C16:0 Incorporation into the sn-2 Position of TAG.

**Fig. 3.**
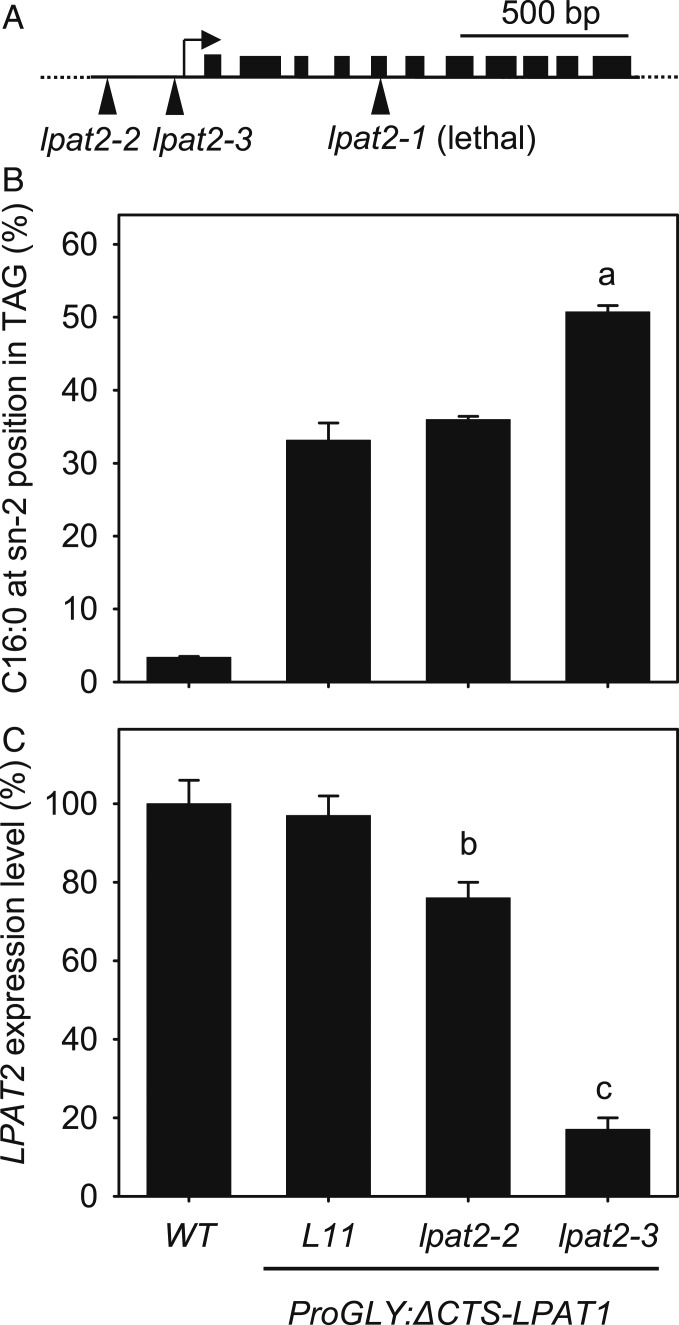
Disruption of ER-resident *LPAT2* increases C16:0 incorporation into the sn-2 position of TAG. (*A*) Diagram of *LPAT2* locus showing positions of T-DNA insertions in mutant alleles. Effect of *lpat2* mutant backgrounds on (*B*) the percentage of C16:0 esterified to the *sn-2* position of TAG, verses sn-1+3, and (*C*) *LPAT2* transcript abundance in seeds expressing *∆CTS-LPAT1*. WT, wild type; L11, homozygous *ProGLY:∆CTS-LPAT1* line. Values are the mean ± SE of measurements made on separate batches of dry seeds in *B* and developing siliques in *C* from 3 plants of each genotype (*n* = 3). *LPAT2* expression was normalized to the geometric mean of 3 reference genes and expressed relative to WT. a, b, and c denote values significantly (*P* < 0.05) different from L11 (ANOVA + Tukey HSD test).

Competition between heterologous and native acyltransferases is one factor that may limit the incorporation of specific fatty acyl groups into TAG (23). We therefore investigated whether ∆CTS-LPAT1-dependent incorporation of C16:0 into the sn-2 position of TAG could be enhanced by disrupting the function of the native ER-resident LPAT; believed to be *LPAT2* in *Arabidopsis* (10) (Fig. 1). The *lpat2-1* null mutant is embryo lethal (10). However, T-DNA insertions in noncoding regions of essential genes can be used to produce viable hypomorphic alleles (24, 25). We therefore isolated 2 T-DNA mutants (*lpat2-2* and *lpat2-3*) with insertions 302 and 139 bp 5′ of the *LPAT2* translational start site (Fig. 3*A*). We then crossed *ProGLY:∆CTS-LPAT1* L11 into each of the new *lpat2* alleles and recovered homozygous seed batches. When we purified TAG from these seed batches and performed positional analysis, we found that the percentage of C16:0 at the sn-2 position had increased from ∼33% in the parental *ProGLY:∆CTS-LPAT1* line to ∼51% in the *lpat2-3* background, whereas the effect in the *lpat2-2* background was not significant (*P* > 0.05) (Fig. 3*B* and *SI Appendix*, Table S3). qRT-PCR analysis showed that *LPAT2* expression is reduced by ∼83% in developing *lpat2-3* siliques, but only by ∼24% in *lpat2-2*. (Fig. 3*B*). These data support the hypothesis that LPAT2 contributes to TAG biosynthesis in *Arabidopsis* seeds (10) and that it competes with ΔCTS-LPAT1. The level of C16:0 enrichment at sn-2 also appears to respond to the strength of *LPAT2* repression and achieving a greater reduction than ∼83% might therefore lead to even stronger enrichment.

### Disruption of PDCT Also Enhances C16:0 Incorporation into the sn-2 Position of TAG.

**Fig. 4.**
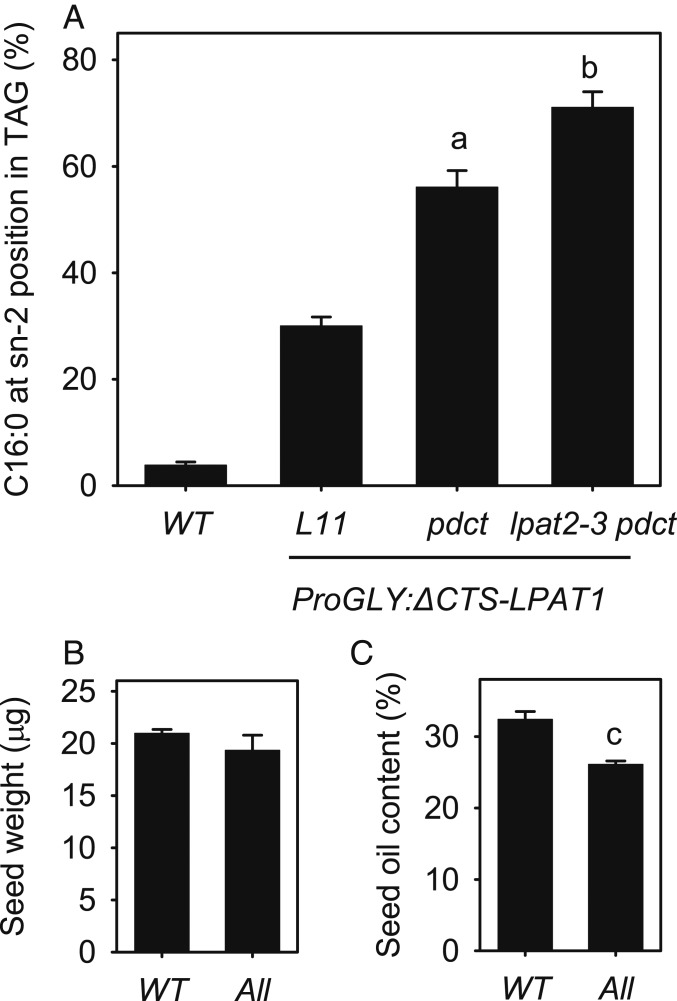
Bypassing flux through PC increases C16:0 incorporation into the sn-2 position of TAG. (*A*) Effect of *pdct* mutant background on percentage of C16:0 esterified to the *sn-2* position of TAG in *ProGLY:∆CTS-LPAT1* and *ProGLY:∆CTS-LPAT1 lpat2-3* seeds. WT, wild type; L11, homozygous *ProGLY:∆CTS-LPAT1* line. (*B*) Seed weight and (*C*) percentage oil content of WT and *ProGLY:∆CTS-LPAT1 lpat2-3 pdct* (All). Values are the mean ± SE of measurements on separate seed batches from between 3 and 6 plants in *A* and 5 plants in *B* and *C* of each genotype (*n* = 3–6). a and b denote values significantly (*P* < 0.05) different from L11 and *pdct*, respectively (ANOVA + Tukey HSD test) and c from WT (2-tailed Student’s *t* test).

In developing *Arabidopsis* seeds >90% of the glycerol backbone in TAG is derived from the membrane lipid phosphatidylcholine (PC), owing to rapid diacylglycerol (DAG)-PC interconversion (26), catalyzed mainly by the plant-specific head group exchange enzyme PC:DAG cholinephosphotransferase (PDCT) (27, 28) (Fig. 1). Although LPAT is responsible for the initial acylation of glycerolipids at sn-2, once these acyl groups are in PC they may be removed and replaced by acyl editing activities (26, 29, 30) (Fig. 1). To determine whether bypassing glycerolipid flux through PC (Fig. 1) might increase ∆CTS-LPAT1-dependent incorporation of C16:0 into the sn-2 position of TAG, we crossed *ProGLY:∆CTS-LPAT1* L11 into the *pdct* (*reduced oleate desaturation1*) mutant (27). When we purified TAG from *ProGLY:∆CTS-LPAT1 pdct* seed batches and performed positional analysis, we found that the percentage of C16:0 at sn-2 had increased from ∼30% in the parental *ProGLY:∆CTS-LPAT1* line to ∼56% in the *pdct* background (Fig. 4*A* and *SI Appendix*, Table S4). These data suggest that a more direct flux of newly made DAG into TAG (28) (Fig. 1) favors C16:0 incorporation and/or retention at the sn-2 position. In WT seeds, it is conceivable that C16:0 entering the sn-2 position of PC might either be edited from it by the action of lysophosphatidylcholine acyltransferase (LPCAT) or a phospholipase A2 (28). Interestingly, Lager et al. (29), have provided in vitro evidence that the reverse activities of *Arabidopsis* LPCAT1 and LPCAT2 can selectively remove certain fatty acyl groups from PC, but C16:0 was not tested. Although rapid DAG-PC interconversion occurs in *Arabidopsis* seeds (26), it is noteworthy that considerable interspecific variation has been reported in this flux (31) and so the effect of PDCT disruption on C16:0 enrichment at the sn-2 of TAG may differ between oilseeds.

### Disruption of LPAT2 and PDCT Has an Additive Effect on Incorporation of C16:0 at sn-2.

To determine whether the combination of reducing LPAT competition and bypassing flux through PC would have an additive effect on ∆CTS-LPAT1-dependent incorporation of C16:0 into the sn-2 position of TAG (Fig. 1), we crossed *ProGLY:∆CTS-LPAT1 lpat2-3* with *ProGLY:∆CTS-LPAT1 pdct*. When we purified TAG from homozygous seed batches and performed positional analysis, we found that the percentage of C16:0 at sn-2 had increased from ∼56% in *ProGLY:∆CTS-LPAT1 pdct* to ∼71% in *ProGLY:∆CTS-LPAT1 lpat2-3 pdct* (Fig. 4*A* and *SI Appendix*, Table S4). The combination of just 3 modifications to the TAG biosynthetic pathway in *Arabidopsis* (i.e., *∆CTS-LPAT1* expression, plus *LPAT2* and *PDCT* suppression) is therefore sufficient to replicate the level of C16:0 enrichment at the sn-2 position (vs. sn-1+3) that is found in HMF (1–3). Analysis of TAG composition in *ProGLY:∆CTS-LPAT1 lpat2-3 pdct* (All) seeds using high resolution/accurate mass (HR/AM) lipidomics (32) also confirmed the presence of C16:0 groups at the sn-2 position, since tripalmitin was 27-fold more abundant than in WT (*SI Appendix*, Fig. S2*A*). By contrast, no dipalmitoyl PC was detected in *ProGLY:∆CTS-LPAT1 lpat2-3 pdct* seeds and molecular species of PC containing one C16:0 group were not increased (*SI Appendix*, Fig. S2*B*). These data suggest that an asymmetrical distribution of saturated and unsaturated fatty acyl groups in PC is maintained in *ProGLY:∆CTS-LPAT1 lpat2-3 pdct* seeds and this may be important to prevent membranes assuming the gel phase at physiological temperatures (33, 34).

### Redistribution of C16:0 Reduces Seed Oil Content, But Not Germination or Establishment.

**Fig. 5.**
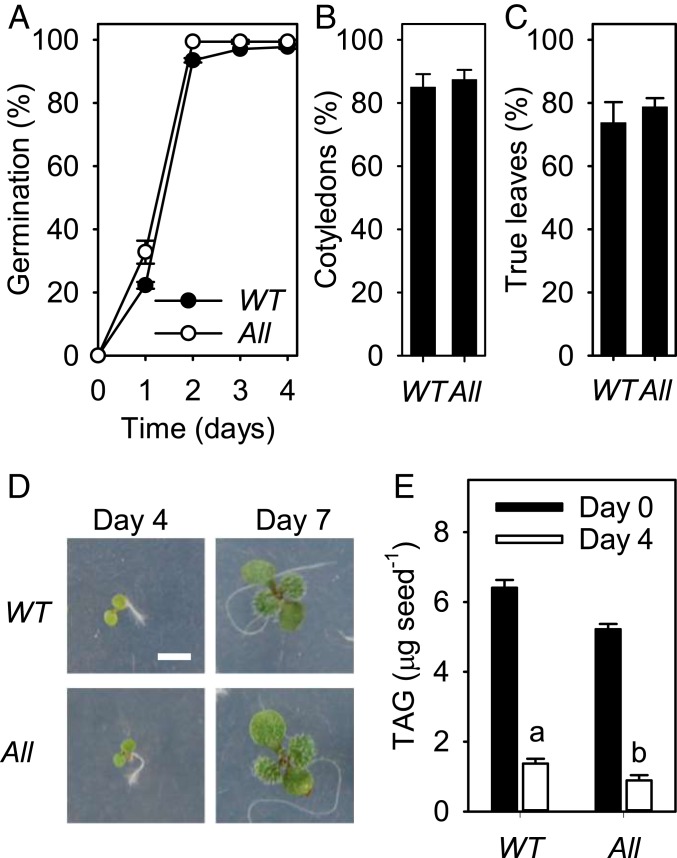
Effect of genetic modifications on seed vigor at 20 °C. Percentage (*A*) seed germination, (*B*) cotyledons expanded by day 4, and (*C*) true leaves developing by day 7. (*D*) Representative images of seedlings with expanded cotyledons and developing true leaves. (*E*) Seed/seedling TAG content at days 0 and 4. WT, wild type; All, *ProGLY:∆CTS-LPAT1 lpat2-3 pdct*. Values are the mean ± SE of measurements made on separate seed batches from 3 plants of each genotype (*n* = 3). In *D*, Scale bar, 2 mm. a and b denote values significantly (*P* < 0.05) different from WT (2-tailed Student’s *t* tests).

Many studies have shown that modifying fatty acyl composition can reduce TAG accumulation in oilseeds and in some cases can also impair seed germination and seedling establishment (35, 36). Our primary objective in this study was not to alter fatty acyl composition per se, but to change the stereoisomeric structure of TAG. To examine the physiological impact of C16:0 enrichment at the sn-2 position of TAG, we compared seed batches from wild type and *ProGLY:∆CTS-LPAT1 lpat2-3 pdct* plants that had been grown together under standard laboratory conditions. We found no significant difference (*P* > 0.05) in seed weight between the 2 genotypes (Fig. 4*B*). However, the fatty acid content of *ProGLY:∆CTS-LPAT1 lpat2-3 pdct* seeds was significantly (*P* < 0.05) lower than that of wild type, when expressed as a percentage of seed weight (Fig. 4*C*). These data suggest that the modifications leading to incorporation of C16:0 into the sn-2 position reduce TAG biosynthetic flux. This finding is consistent with previous studies in which seed TAG composition has been modified either using genetic engineering or mutant breeding methods (35, 36). In warm conditions (20 °C), *ProGLY:∆CTS-LPAT1 lpat2-3 pdct* seed germination, scored as radicle emergence (Fig. 5*A*) and seedling establishment, scored as cotyledon expansion (Fig. 5*B*) and true leaf development (Fig. 5*C*), did not appear to be significantly (*P* < 0.05) impaired, relative to wild type. TAG breakdown also was not impeded in *ProGLY:∆CTS-LPAT1 lpat2-3 pdct* seeds following germination in warm conditions (Fig. 5*D*), and this contrasts with some studies where seeds have been modified to incorporate uncommon fatty acyl groups into TAG (35). In cool conditions (10 °C), *ProGLY:∆CTS-LPAT1 lpat2-3 pdct* seed germination and seedling establishment also appeared not to be significantly (*P* < 0.05) impaired, relative to wild type (*SI Appendix*, Fig. S3). Finally, although *ProGLY:∆CTS-LPAT1 lpat2-3 pdct* carries a hypomorphic allele of the essential gene *LPAT2* (10) (Fig. 3), this does not appear to adversely affect growth and morphology at the rosette stage (*SI Appendix*, Fig. S4).

## Conclusions

In this study we show that the TAG biosynthetic pathway in plants can be engineered so that the stereoisomeric structure of seed storage oil is altered to mimic that of HMF, with >70% of C16:0 concentrated at the middle (sn-2 or β) position on the glycerol backbone. There is mounting evidence that this configuration is beneficial for infant nutrition (1, 3, 6), but it has not been found to occur naturally in vegetable fats where C16:0 is virtually excluded from the sn-2 position (4, 5, 9). Many infant formulas contain HMFS that are made by restructuring vegetable fats using enzyme-based catalysis, but they are relatively costly to produce; particularly for the manufacture of true mimetics with >70% of C16:0 at the sn-2 position (1, 7). Translation of our technology from the model species *Arabidopsis* to an oilseed crop might conceivably provide a cheaper and more sustainable source of HMFS for infant formula, but further research would be required to test this supposition. If HMFS could be obtained directly from a vegetable source, this would abrogate the need for enzyme-based catalysis. The infant formula market is currently estimated to use nearly half a million metric tons of vegetable-derived fat per year. Several oilseed crops may be considered as possible hosts for HMFS production, and it is noteworthy that conventional sunflower (*Helianthus annus*) and genetically modified oilseed rape (*B. napus*) varieties have already been developed that have the appropriate fatty acyl composition (37, 38). Even an oilseed crop with more modest C16:0 enrichment at the sn-2 position than we have achieved here may still be desirable since clinical trials have reported benefits with as little as 43% of C16:0 at the sn-2 position (1, 3, 6) and product surveys have found that this level of enrichment is common in infant formulas that are supplemented with HMFS (1).

## Materials and Methods

Detailed descriptions of plant material and growth conditions, cloning, and Agrobacterium-mediated transformation, microscopy, mutant genotyping, lipid analysis, qRT-PCR analysis of gene expression, germination and seedling establishment assays, and statistical analysis are provided in *SI Appendix*, *SI Materials and Methods*. Primers used are listed in *SI Appendix*, Table S5.

## Supplementary Material

Supplementary File
