# Supplementary material for: Engineering the stereoisomeric structure of seed oil to mimic human milk fat
Source: Proc Natl Acad Sci U S A. 2019 Sep 30;116(42):20947–52. doi: 10.1073/pnas.1907915116 (PMC6800330; doi:10.1073/pnas.1907915116)
Supplement: Supplementary File [file pnas.1907915116.sapp.pdf]

PNAS
www.pnas.org

Supplementary Information for

Engineering the stereoisomeric structure of seed oil to mimic human milk fat

Harrie van Erp, Fiona M. Bryant, Jose Martin-Moreno, Louise V. Michaelson, Govindprasad Bhutada, Peter J Eastmond

Peter J Eastmond
Email: peter.eastmond@rothamsted.ac.uk

This PDF file includes:

Supplementary text
Figures S1 to S4
Tables S1 to S5
SI References

Supplementary Information Text

SI Materials and Methods

Plant material and growth conditions

Arabidopsis thaliana wild type (Col-0) and *LPAT2* (At3g57650) T-DNA insertion lines SALK_108026 (*lpat2-2*) and SALK_004681 (*lpat2-3*) (1) were obtained from the European Arabidopsis Stock Centre (University of Nottingham, UK). The *pdct* (*rod1*) mutant has been described previously (2). Seeds were surface sterilized, stratified at 4°C for two days and germinated on agar plates containing ½ strength MS medium (Sigma-Aldrich) pH 5.7. Seedlings were transplanted to 7 cm^2 pots containing Levington F2 compost and grown in a chamber set to a 16-h light (22 °C)/8-h dark (16 °C) cycle, with a light intensity of 250 µmol m^{-2} s^{-1}. The plants were bagged individually at the onset of flowering and the seeds were harvested at maturity.

Cloning and transformation

RNA was isolated from *Brassica napus* (cv. Kumily) seedlings and DNAse treated using the RNAeasy Plant Mini kit (Qiagen). RNA was reverse transcribed into cDNA with the SuperScript III Reverse Transcriptase kit (ThermoFisher Scientific). *B. napus LPAT1* (GenBank: AF111161) lacking the chloroplast targeting sequence (CTS) was amplified by PCR with KOD DNA polymerase (Merck) using primer pair P1+P2 (Table S5). The resulting PCR product was purified with the QIAquick Gel Extraction Kit (Qiagen). For localisation studies, ∆*CTS-LPAT1* was cloned behind

www.pnas.org/cgi/doi/10.1073/pnas.1907915116

Red Fluorescent Protein (RFP) in the pK7WGR2 vector (Vlaams Institute for Biotechnology). The PCR product was cloned in the pENTR™/D-TOPO™ vector (Thermo Fisher Scientific), sequenced (Fig. S1) and recombined into the pK7WGR2 vector using the Gateway™ LR Clonase™ II Enzyme mix (Thermo Fisher Scientific). Δ*CTS-LPAT1* was cloned in the pBinGlyRed3 vector in between the soybean glycinin-1 (GLY) promoter and terminator for seed specific expression (3). Δ*CTS-LPAT1* was PCR-amplified from the pENTR-D-TOPO vector using KOD DNA polymerase and primer pair P3+P4. The PCR product was gel purified and digested with EcoRI and XbaI. The pBinGlyRed3 vector was also digested with EcoRI and XbaI, alkaline phosphatase treated (Promega), gel purified and Δ*CTS-LPAT1* was ligated into the vector using T4 DNA ligase (NEB). Heat shock was used to transform the vectors into *Agrobacterium tumefaciens* strain GV3101 and Arabidopsis transformation was then carried out using the floral-dip method (4). T1 seeds expressing the selectable marker were identified under a Leica M205 FA microscope using the DsRed filter.

Transient expression in *Nicotiana benthamiana* and imaging
Transient expression in *Nicotiana benthamiana* leaves was carried out as described by Wood et al., (5) using *A. tumefaciens* cultures transformed with vectors harbouring *Pro35S:RFP-ΔCTS-LPAT1*, *Pro35S:m-GFP5-ER* or *Pro35S:p19*. Cultures were hand-infiltrated into leaves and the inoculated plants were left for 48 h. *N. benthamiana* leaves were then mounted in water on a Zeiss LSM 780 laser scanning confocal microscope under an Apochromat 63x/1.20 W Korr M27 objective. GFP was excited at a wavelength of 488 nm and RFP at 561 nm. Filters with an emission band at 473-551 nm were used for detection.

Genotyping
Genomic DNA was isolated using the DNeasy Plant Mini Kit (Qiagen). Homozygous T-DNA insertional mutants were identified by PCR (1) using the Promega PCR Master Mix (Promega). The following primer pairs were used for genotyping *lpat2-2* (WT: P5+P6 or T-DNA: P5+P7) and *lpat2-3* (WT: P8+P9 or T-DNA: P8+P7). *PDCT* was genotyped by sequencing PCR products amplified with primer pair P10+P11 spanning the site of the point mutation (2). *ProGLY:ΔCTS-LPAT1* was genotyped by PCR using primer pair P12+P13 spanning *ProGLY* and Δ*CTS-LPAT1*.

Lipid analysis
Total lipids were extracted from seeds and seedlings and TAG was purified as described previously (6). TAG stereochemical analysis was performed by lipase digestion following the method described previously (7), except that 2-monoacylglycerols were separated by thin layer chromatography (Silica gel 60, 20 x 20 cm; Sigma-Aldrich/Merck) using hexane:diethylether:acetic acid (35:70:1.5, v/v/v) (8). Fatty acyl groups present in whole seeds and purified lipid fractions were trans-methylated and quantified by gas chromatography (GC) coupled to flame ionization detection, as described previously (9), using a 7890A GC system fitted with DB-23 columns (30 m x 0.25 mm i.d. x 0.25 µm) (Agilent Technologies).
TAG and PC molecular species composition were analysed by high resolution / accurate mass (HR/AM) lipidomics (10-12) using a Vanquish - Q Exactive Plus UPLC-MS/MS system (Thermo Fisher Scientific). Work flow consisted of using total lipids purified at 3 µg/µl and diluted 1 in 100 in chloroform:methanol (1:1, v/v). Internal tripalmitin standard (0.857µM) was added and 20µl injected into the UPLC. Lipids were separated using a Accucore C18 (2.1 x 150 mm, 2.6 mm) column (Thermo Fisher Scientific) at 35°C with autosampler tray temperature, 10°C flow rate at 400 µl min^{-1}. Mobile phase: A = 10 mM ammonium formate in 50% acetonitrile + 0.1% formic acid, B = 2 mM ammonium formate in acetonitrile:propan-2-ol:water (10:88:2 v/v) + 0.02% formic acid. Elution gradient ran for 28 minutes from 35% B at start to 100% at 24 mins. Thermo Q Exactive HESI II probe conditions, sweep plate in use probe position in C. Conditions were adjusted for separate positive and negative runs, running samples in a single polarity resulted in more identifications. LC/MS at 140K resolution and data-independent HCD MS2 experiments (35K resolution) were performed in positive and negative ion modes. Full Scan @ 140,000 resolution m/z 150-1200 Top 15 most abundant MS/MS @ 35,000 resolution using an isolation window of 1 m/z, maximum integration time of 75 ms and dynamic exclusion window of 8s. The stepped collision energy was 25, 30, 40 eV replacing 25 with 30 eV negative ion mode. Sheath gas set to 60, Aux gas 20, sweep

gas 1 spray voltage 3.2 KV in positive ion mode with small adjustments in negative ion mode, capillary temperature 320 and aux gas heater set to 370°C. LipidSearch 4.2 experimental workflow (Thermo Fisher Scientific) was used for lipid characterization and potential lipid species were identified separately from positive or negative ion adducts. The data for each biological replicate were aligned within a chromatographic time window by combining the positive and negative ion annotations and merging these into a single lipid annotation.

qRT-PCR analysis

DNAse-treated total RNA was isolated from developing siliques as described by Mendes et al., (13). The synthesis of single stranded cDNA was carried out using SuperScriptTM II RNase H- reverse transcriptase from Invitrogen Ltd. (Paisley, UK). Quantitative (q)-PCR was performed as described previously (13), except that *LPAT2* and Δ*CTS-LPAT1* expression were normalized to the geometric mean of three reference genes (*UBQ5*, *EF-1α* and *ACT8*) that were selected owing to their stable expression over the course of seed development (14). Primer pairs P14+P15, P16+P17, P18+P19, P20+P21 and P22+P23, were used for *LPAT2*, Δ*CTS-LPAT1*, *UBQ5*, *EF-1α* and *ACT8*, respectively.

Seed germination and establishment assays

Around 50 seeds from each plant were surface sterilized, sown on a ½ MS agar plate, stratified at 4°C for two days and transferred to a growth chamber set to 20°C or 10°C, 16h light/8h dark, PPFD = 150 µmol m^{-2} s^{-1}. Germination (radicle emergence), expanded cotyledons and expanded true leaves were scored visually under a dissecting stereomicroscope. Seeds and seedlings were also collected at zero and four days after stratification for lipid analysis.

Statistical analysis

All experiments were carried out using between three and six biological replicates and the data are presented as the mean values ± standard error of the mean (SE). For statistical analysis we either used one-way analysis of variance (ANOVA) with post-hoc Tukey HSD (Honestly Significant Difference) tests, or two-tailed Student's *t*-tests.

```
>DNA sequence of ΔCTS-LPAT1
ATGTCGGATCTTTCAGGAGCTGCAACCCCTGAATCTACTTATCCAGAACCAGAGATTAAGTTGAGCTCAAG
ACTCAGAGGGATATGCTTTTGTCTCGTTGCTGGCGTTTCCGCCATTGTTCTCATCGTCCTGATGATCACTG
GCCATCCTTTCGTCCTTCTATTTGATCGTTACAGGAGGAAGTTCCATCACTTCATTGCTAAGCTCTGGGCT
TCCATAAGCATCTACCCGTTTTACAAAACCGACATCCAAGGTTTGGAGAATCTGCCATCATCAGACACTCC
TTGTGTGTACGTTTCGAACCACCAGAGTTTTCTGGATATATACACACTTCTCAGCCTTGGCCAAAGCTATA
AGTTCATCAGCAAGACAGGGATATTCGTTATTCCTGTCATCGGTTGGGCTATGTCCATGATGGGGGTTGTT
CCCTTGAAGAGGATGGACCCAAGAAGCCAAGTGGATTGCTTAAAACGCTGCATGGAACTAGTGAAGAAGGG
AGCTTCCGTCTTTTTCTTCCCAGAGGGAACGAGGAGTAAGGATGGTCGGTTAGGTCCTTTCAAGAAAGGGG
CTTTTACGATAGCAGCTAAGACAGGAGTTCCAGTGGTGCCAATAACGCTGATGGGAACAGGGAAGATCATG
CCGACGGGTAGTGAAGGTATACTGAATCATGGGGATGTGAGAGTGATCATCCACAAGCCGATATATGGAAG
CAAAGCTGATCTTCTTTGCGATGAGGCTAGAAACAAGATAGCTGAATCTATGAATCTCTTGAGTTGA

>Amino acid sequence of ΔCTS-LPAT1
MSDLSGAATPESTYPEPEIKLSSRLRGICFCLVAGVSAIVLIVLMITGHPFVLLFDRYRRKFHHFIAKLWA
SISIYPFYKTDIQGLENLPSSDTPCVYVSNHQSFLDIYTLLSLGQSYKFISKTGIFVIPVIGWAMSMMGVV
PLKRMDPRSQVDCLKRCMELVKKGASVFFFPEGTRSKDGRLGPFKKGAFTIAAKTGVPVVPITLMGTGKIM
PTGSEGILNHGDVRVIIHKPIYGSKADLLCDEARNKIAESMNLLS
```

Fig. S1. Sequence of Δ*CTS-LPAT1*.

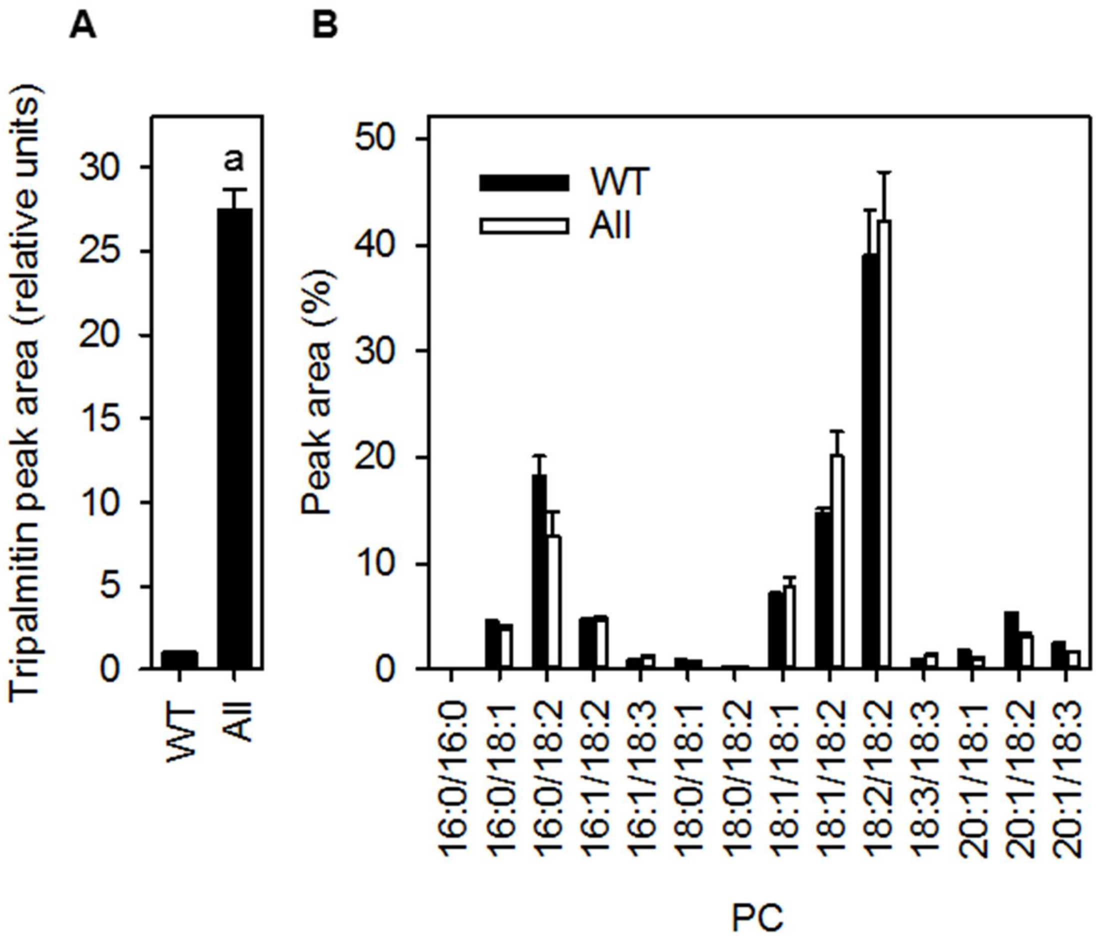

Fig. S2. Effect of genetic modifications on seed lipid composition. (*A*) Tripalmitin content and (*B*) phosphatidylcholine (PC) composition. WT = wild type; All = *ProGLY:ΔCTS-LPAT1 lpat2-3 pdct*. Values are the mean ±SE of measurements made on separate seed batches from five plants of each genotype (n = 5). a denotes a value significantly (P < 0.05) different from WT (two-tailed Student's *t* tests).

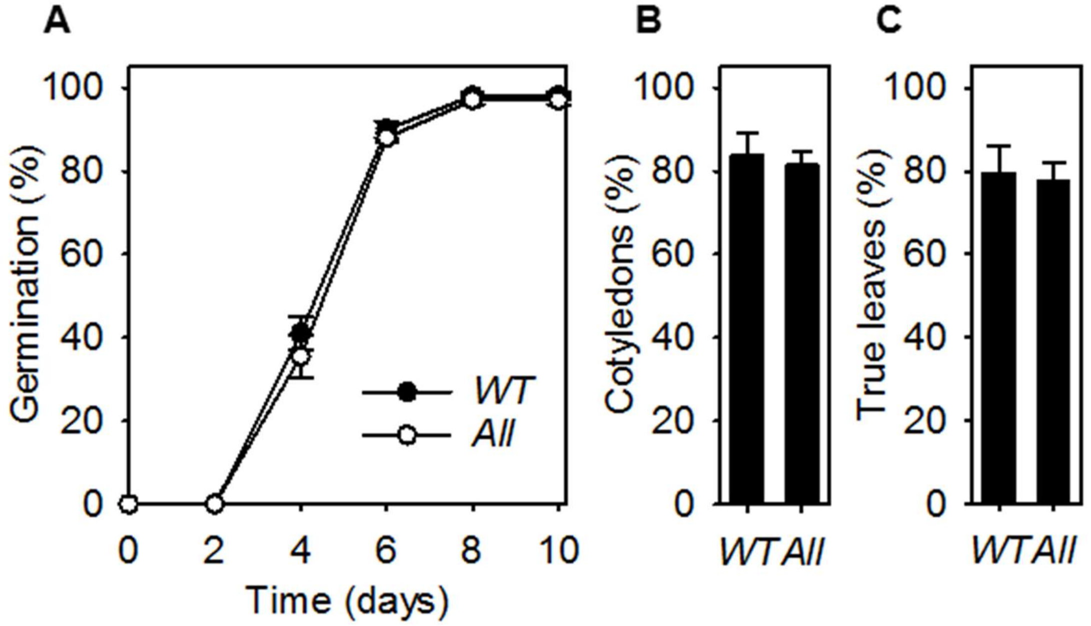

Fig. S3. Effect of genetic modifications on seed vigour at 10ºC. Percentage (*A*) seed germination, (*B*) cotyledons expanded by day 14 and (*C*) true leaves developing by day 28. WT = wild type; All = *ProGLY:ΔCTS-LPAT1 lpat2-3 pdct*. Values are the mean ±SE of measurements made on separate seed batches from three plants of each genotype (n = 3). All values are not significantly (P > 0.05) different from WT (ANOVA + Tukey HSD test in *A* and two-tailed Student's t tests in *B* and *C*).

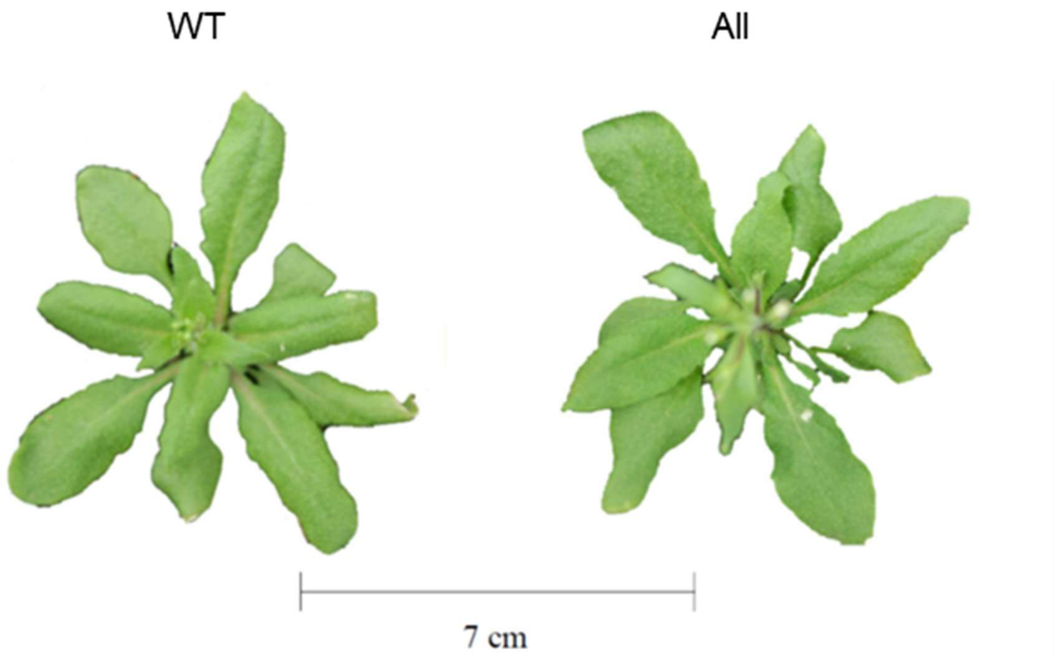

Fig. S4. Images of wild type (WT) and *ProGLY:ΔCTS-LPAT1 lpat2-3 pdct* (All) plants at onset of flowering. Scale bar = 7 cm.

Table S1. Total fatty acid composition of seed batches from individual wild type (WT) and segregating T2 *ProGLY:ΔCTS-LPAT1* lines (L1-42). Asterisk denotes single copy lines taken forward for further analysis.

Line	Fatty acid content (%)					
	16:0	18:0	18:1	18:2	18:3	20:1
WT 1	8.00	2.78	13.90	30.90	22.67	22.01
WT 2	8.22	2.89	13.98	30.61	22.82	21.74
WT 3	8.25	2.89	13.55	30.63	23.06	21.88
WT 4	8.01	2.92	14.42	30.68	22.47	21.75
WT 5	8.16	2.60	13.91	31.08	22.23	22.27
L1	7.83	3.67	18.16	31.16	18.99	20.19
L2	9.27	3.42	17.90	31.03	19.03	19.34
L3	8.28	3.20	16.88	31.52	19.19	20.93
L4	8.92	3.99	17.69	30.77	19.33	19.31
L5	8.15	4.06	18.24	32.40	18.13	19.02
L6*	9.97	3.55	17.16	30.69	20.03	18.60
L7	7.84	3.16	17.09	31.15	20.44	20.33
L8	7.72	3.21	17.71	31.37	20.14	19.86
L9	8.47	3.43	17.85	30.87	19.51	19.88
L10	9.62	3.50	18.78	30.25	19.99	17.86
L11*	10.67	3.44	12.91	33.47	21.74	17.77
L13	8.63	2.50	15.27	31.12	21.88	20.61
L14	7.65	2.97	17.26	30.70	21.25	20.18
L15	8.43	3.42	17.09	30.41	20.35	20.30
L16	7.92	3.34	18.25	31.03	19.90	19.55
L17	9.73	3.05	15.59	31.22	21.84	18.58
L18	7.95	3.44	18.56	30.79	19.82	19.44
L19	8.22	3.38	17.62	30.97	20.17	19.65
L20	7.62	3.61	18.74	31.27	20.16	18.60
L21	7.77	3.47	17.55	30.67	20.70	19.84
L22	8.20	3.37	19.47	31.89	17.86	19.21
L23	7.81	3.12	18.98	30.55	19.32	20.22
L24	7.64	3.67	18.94	30.96	19.11	19.69
L25	9.06	2.64	12.92	33.09	21.56	20.74
L26	9.64	2.79	20.64	28.78	19.87	18.29
L27	7.83	3.50	18.73	31.22	19.15	19.56
L28	7.62	3.59	19.73	31.55	18.56	18.96
L29	7.45	3.72	19.45	31.55	18.94	18.89
L30*	9.88	3.57	19.36	30.37	19.14	17.68
L31	7.60	3.53	18.40	31.11	19.59	19.77
L32	9.19	3.64	18.45	31.83	18.60	18.30
L33	7.71	3.76	18.37	31.26	19.65	19.25
L34	7.56	3.62	19.03	30.69	19.46	19.64
L35	7.36	3.61	20.01	31.15	18.32	19.55
L36	8.81	3.64	18.75	31.23	18.99	18.58
L37	7.55	3.78	19.42	31.55	18.46	19.24
L38	7.34	3.99	23.01	30.95	15.89	19.82
L39	8.07	3.70	19.15	31.74	18.50	18.84
L41	9.59	3.84	18.05	31.58	19.13	17.82
L42	7.80	3.46	18.69	30.51	21.11	19.43

Table S2. Total and *sn-2* fatty acyl composition of TAG from wild type (WT) and homozygous *ProGLY:ΔCTS-LPAT1* seeds.

Line	Fatty acid content (%)								
	16:0	18:0	18:1	18:2	18:3	20:0	20:1	20:2	22:1
Total									
WT 1	8.23	4.17	17.56	28.10	15.87	1.97	21.02	1.63	1.36
WT 2	8.14	4.05	18.19	28.06	16.31	2.01	20.21	1.62	1.42
WT 3	8.31	3.99	16.46	28.77	16.48	2.10	20.53	1.83	1.53
L30 1	9.39	3.08	17.75	29.03	16.15	1.90	19.49	1.56	1.64
L30 2	10.26	3.27	19.08	28.37	15.43	1.95	18.89	1.37	1.38
L30 3	8.53	3.24	18.90	28.24	15.23	1.94	20.80	1.48	1.64
L6 1	8.55	3.08	17.70	28.49	16.28	1.85	20.74	1.64	1.67
L6 2	9.58	3.26	17.94	28.43	16.32	1.95	19.54	1.53	1.53
L6 3	8.04	2.77	17.50	29.24	16.26	1.70	20.94	1.74	1.74
L11 1	7.15	3.15	16.97	27.94	18.17	2.43	20.75	1.93	1.50
L11 2	7.68	3.29	16.20	26.98	15.14	2.14	24.81	2.19	1.30
L11 3	7.57	3.27	15.79	26.20	18.70	2.13	21.95	2.46	1.93
sn-2									
WT 1	0.53	0.25	18.07	53.05	28.10	0.00	0.00	0.00	0.00
WT 2	0.33	0.24	18.49	52.52	28.43	0.00	0.00	0.00	0.00
WT 3	0.60	0.53	16.70	54.30	27.87	0.00	0.00	0.00	0.00
L30 1	9.22	0.30	16.65	49.81	24.03	0.00	0.00	0.00	0.00
L30 2	7.50	0.64	19.15	49.70	23.01	0.00	0.00	0.00	0.00
L30 3	10.01	0.64	17.79	48.73	22.83	0.00	0.00	0.00	0.00
L6 1	10.17	0.68	15.88	49.23	24.04	0.00	0.00	0.00	0.00
L6 2	9.21	0.26	17.24	48.90	24.40	0.00	0.00	0.00	0.00
L6 3	8.59	0.75	15.53	50.71	24.42	0.00	0.00	0.00	0.00
L11 1	8.49	0.37	14.99	48.02	28.13	0.00	0.00	0.00	0.00
L11 2	9.77	n.d.	14.34	48.84	27.06	0.00	0.00	0.00	0.00
L11 3	7.88	1.17	14.76	47.08	29.12	0.00	0.00	0.00	0.00

Table S3. Total and *sn-2* fatty acyl composition of TAG from wild type (WT) and homozygous *ProGLY:ΔCTS-LPAT1* seeds containing *lpat2* mutant alleles.

Line	Fatty acid content (%)								
	16:0	18:0	18:1	18:2	18:3	20:0	20:1	20:2	22:1
Total									
WT 1	7.39	3.51	18.41	29.26	16.08	2.00	20.41	1.70	1.25
WT 2	7.53	3.44	17.51	29.13	16.69	2.02	20.32	1.80	1.56
WT 3	7.96	3.41	17.68	29.31	16.91	1.92	19.64	1.72	1.46
L11 1	8.58	3.45	13.18	26.68	19.35	2.73	22.10	2.11	1.82
L11 2	9.03	2.79	15.48	27.50	20.67	1.90	19.16	1.84	1.63
L11 3	8.49	2.87	15.33	27.07	20.89	1.96	20.00	1.89	1.50
L11 *lpat2-2* 1	8.69	3.29	15.66	26.67	19.76	2.10	20.58	1.82	1.44
L11 *lpat2-2* 2	9.28	3.31	15.51	27.53	19.74	1.94	19.49	1.80	1.40
L11 *lpat2-2* 3	8.58	2.77	13.18	29.05	20.04	2.30	20.56	2.02	1.50
L11 *lpat2-3* 1	9.04	3.17	18.75	27.28	15.90	1.64	21.58	1.28	1.36
L11 *lpat2-3* 2	9.08	3.09	18.33	27.50	16.11	1.63	21.37	1.39	1.50
L11 *lpat2-3* 3	8.20	3.09	18.04	27.90	15.18	1.72	22.84	1.51	1.53
sn-2									
WT 1	0.78	0.53	18.54	54.22	25.93	0.00	0.00	0.00	0.00
WT 2	0.78	0.52	17.22	54.11	27.37	0.00	0.00	0.00	0.00
WT 3	0.75	0.53	17.38	53.89	27.44	0.00	0.00	0.00	0.00
L11 1	9.56	0.68	14.68	46.54	28.53	0.00	0.00	0.00	0.00
L11 2	7.83	0.00	14.05	46.24	31.88	0.00	0.00	0.00	0.00
L11 3	8.10	0.00	14.43	46.01	31.46	0.00	0.00	0.00	0.00
L11 *lpat2-2* 1	9.56	0.37	15.63	45.83	28.61	0.00	0.00	0.00	0.00
L11 *lpat2-2* 2	9.79	0.65	14.12	45.05	30.39	0.00	0.00	0.00	0.00
L11 *lpat2-2* 3	9.29	0.35	13.62	49.00	27.74	0.00	0.00	0.00	0.00
L11 *lpat2-3* 1	13.31	0.60	16.58	46.78	22.73	0.00	0.00	0.00	0.00
L11 *lpat2-3* 2	13.88	0.95	15.53	46.81	22.82	0.00	0.00	0.00	0.00
L11 *lpat2-3* 3	12.82	1.30	16.70	48.02	21.15	0.00	0.00	0.00	0.00

Table S4. Total and *sn-2* fatty acyl composition of TAG from wild type (WT) and homozygous *ProGLY:ΔCTS-LPAT1* seeds containing *pdct* and *lpat2-3* mutant alleles.

Line	Fatty acid content (%)								
	16:0	18:0	18:1	18:2	18:3	20:0	20:1	20:2	22:1
Total									
WT 1	7.84	3.38	17.20	28.95	16.89	2.02	20.23	1.82	1.69
WT 2	7.89	3.43	17.97	29.09	16.58	1.90	19.82	1.69	1.63
WT 3	7.65	3.77	15.95	28.91	17.43	2.31	20.54	1.85	1.59
WT 4	7.67	3.80	17.03	28.57	16.88	2.18	20.58	1.74	1.54
WT 5	8.14	3.60	17.39	28.98	16.12	2.00	20.42	1.74	1.61
WT 6	7.96	3.35	15.79	29.27	17.67	1.89	20.59	1.73	1.75
L11 1	9.06	3.48	15.98	28.47	18.30	0.00	21.47	1.74	1.51
L11 2	6.47	3.08	15.13	27.52	19.77	2.18	22.49	1.52	1.57
L11 3	10.16	3.37	19.48	27.91	15.94	1.88	18.67	1.26	1.33
L11 4	7.66	3.15	38.93	13.00	13.24	1.72	20.54	0.39	1.37
L11 5	8.35	3.03	36.89	14.26	14.30	1.64	19.70	0.42	1.41
L11 6	10.34	3.27	18.30	27.89	16.78	1.85	18.85	1.37	1.35
L11 *pdct* 1	9.63	3.06	33.88	13.74	15.46	1.73	19.38	1.29	1.82
L11 *pdct* 2	8.80	3.15	34.60	12.20	14.33	1.86	20.56	1.64	2.85
L11 *pdct* 3	8.75	2.91	34.46	14.41	14.33	1.73	21.16	0.55	1.70
L11 *lpat2-3 pdct* 1	10.44	2.92	35.40	15.08	14.48	1.41	18.44	0.46	1.38
L11 *lpat2-3 pdct* 2	10.88	2.84	35.97	15.31	13.77	1.27	18.25	0.53	1.19
L11 *lpat2-3 pdct* 3	8.77	2.95	36.57	13.57	12.28	1.48	22.96	0.00	1.41
L11 *lpat2-3 pdct* 4	9.36	2.86	35.16	13.55	13.69	1.55	21.59	0.62	1.60
L11 *lpat2-3 pdct* 5	9.50	3.07	34.06	13.88	14.43	1.64	21.47	0.63	1.33
sn-2									
WT 1	0.67	0.41	17.41	53.86	27.65	0.00	0.00	0.00	0.00
WT 2	0.70	0.52	17.67	54.01	27.11	0.00	0.00	0.00	0.00
WT 3	1.48	1.20	15.30	52.76	29.26	0.00	0.00	0.00	0.00
WT 4	0.85	0.85	16.42	53.22	28.67	0.00	0.00	0.00	0.00
WT 5	0.80	0.81	16.33	54.37	29.78	0.00	0.00	0.00	0.00
WT 6	0.91	0.86	14.79	53.66	28.43	0.00	0.00	0.00	0.00
L11 1	8.54	0.56	14.09	48.35	28.46	0.00	0.00	0.00	0.00
L11 2	6.88	0.33	12.68	48.70	31.41	0.00	0.00	0.00	0.00
L11 3	7.90	0.88	18.17	48.31	24.74	0.00	0.00	0.00	0.00
L11 4	6.79	0.39	47.28	23.74	21.80	0.00	0.00	0.00	0.00
L11 5	8.00	0.00	43.04	25.61	23.36	0.00	0.00	0.00	0.00
L11 6	8.03	0.79	17.28	48.36	25.53	0.00	0.00	0.00	0.00
L11 *pdct* 1	16.16	0.00	36.12	23.39	24.32	0.00	0.00	0.00	0.00
L11 *pdct* 2	16.24	0.59	38.52	21.43	23.23	0.00	0.00	0.00	0.00
L11 *pdct* 3	13.35	0.00	38.81	25.71	22.14	0.00	0.00	0.00	0.00
L11 *lpat2-3 pdct* 1	19.54	0.00	36.33	23.93	20.20	0.00	0.00	0.00	0.00
L11 *lpat2-3 pdct* 2	22.57	0.38	35.99	23.04	18.02	0.00	0.00	0.00	0.00
L11 *lpat2-3 pdct* 3	20.36	0.41	40.58	21.56	17.09	0.00	0.00	0.00	0.00
L11 *lpat2-3 pdct* 4	21.18	0.00	37.97	21.86	18.99	0.00	0.00	0.00	0.00
L11 *lpat2-3 pdct* 5	20.30	0.00	37.53	22.35	19.82	0.00	0.00	0.00	0.00

Table S5. Primers used in study

**P1:**	**5’-CACCATGTCGGATCTTTCAGGAGCTG-3’**
**P2**	**5'-TCAACTCAAGAGATTCATAGATTCA-3'**
**P3**	**5'-CGGAATTCATGTCGGATCTTTCAGGAGC-3'**
**P4**	**5'-GCTCTAGATCAACTCAAGAGATTCATAGATTC-3'**
**P5:**	**5’-GTTGATTGAATCGAGGAAGGA-3’**
**P6:**	**5’-CTTTTTACCACATGCAAAGGG-3’**
**P7:**	**5’-CCATTTTGGTGGTCGTCTAT-3’**
**P8:**	**5’-CAGAAAAATTAACCGGGTGGT-3’**
**P9:**	**5’-CAGCTGAAACCGACGTCTCT-3’**
**P10:**	**5’-GGTCACGCGCTCTTTGAATC-3’**
**P11:**	**5’-GTAGCACCCAAGGCTTCCA-3’**
**P12:**	**5’-GCCAGTGATCATCAGGACG-3’**
**P13:**	**5’-GCGTGGACCGCTTGCTGCAACT-3’**
**P14:**	**5’-CTGGTGGGCTGGAGTTAAGA-3’**
**P15:**	**5’-AGAATCCATCCCACAAGCCA-3’**
**P16:**	**5’-TGTCATCGGTTGGGCTATGT-3’**
**P17:**	**5’-CATCAGCGTTATTGGCACCA-3’**
**P18:**	**5’-GACGCTTCATCTCGTCC-3’**
**P19:**	**5’-CCACAGGTTGCGTTAG-3’**
**P20:**	**5’-TCCAGCTAAGGGTGCC-3’**
**P21:**	**5’-GGTGGGTACTCGGAGA-3’**
**P22:**	**5’-GAATTACCCGACGGACA-3’**
**P23:**	**5’-ACGGTCTGCAATACCT-3’**

SI References

1. J. M. Alonso *et al.,* Genome-wide insertional mutagenesis of *Arabidopsis thaliana*. *Science* **301**, 653-657 (2003).
2. C. Lu, Z. Xin, Z. Ren, M. Miquel, J. Browse, An enzyme regulating triacylglycerol composition is encoded by the ROD1 gene of Arabidopsis. *Proc. Natl. Acad. Sci. U.S.A.* **106**, 18837-18842 (2009).
3. C. Zhang *et al.,* Genetic and biochemical basis for alternative routes of tocotrienol biosynthesis for enhanced vitamin E antioxidant production. *Plant J.* **73**, 628–639 (2013).
4. S. J. Clough, A. F. Bent, Floral dip: a simplified method for Agrobacterium-mediated transformation of Arabidopsis thaliana. *Plant J.* **16**, 735–743 (1998).
5. C. C. Wood *et al.,* A leaf-based assay using interchangeable design principles to rapidly assemble multistep recombinant pathways. *Plant Biotech. J.* **7**, 914-924 (2009).
6. A. A. Kelly *et al.,* The SUGAR-DEPENDENT1 lipase limits triacylglycerol accumulation in vegetative tissues of Arabidopsis. *Plant Physiol.* **162**, 1282-1289 (2013).
7. H. van Erp, P. D. Bates, J. Burgal, J. Shockey, J. Browse, Castor phospholipid:diacylglycerol acyltransferase facilitates efficient metabolism of hydroxy fatty acids in transgenic Arabidopsis. *Plant Physiol.* **155**, 683-693 (2011).
8. P. D. Bates, J. Browse, The pathway of triacylglycerol synthesis through phosphatidylcholine in Arabidopsis produces a bottleneck for the accumulation of unusual fatty acids in transgenic seeds. *Plant J.* **68**, 387-399 (2011).
9. H. van Erp, A. A. Kelly, G. Menard, P. J. Eastmond, Multigene engineering of triacylglycerol metabolism boosts seed oil content in Arabidopsis*. Plant Physiol.* **165**, 30-36 (2014).
10. T. Yamada *et al.,* Development of a lipid profiling system using reverse-phase liquid chromatography coupled to high-resolution mass spectrometry with rapid polarity switching and an automated lipid identification software. *J. Chromatogr. A* **1292**, 211-218 (2013).
11. M. Narvaez-Rivas, G. Zhang, Comprehensive untargeted lipidomic analysis using core-shell C30 particle column and high field orbitrap mass spectrometer. *J. Chromatogr. A* **1440**, 123-134 (2016).
12. N. Turner *et al.,* A selective inhibitor of ceramide synthase 1 reveals a novel role in fat metabolism, *Nat. Comm.* **9**, 3165 (2018).
13. A. Mendes *et al.,* bZIP67 regulates the omega-3 fatty acid content of Arabidopsis seed oil by activating FATTY ACID DESATURASE 3. *Plant Cell* **25**, 3104-3116 (2013).
14. L. Gutierrez *et al.,* The lack of a systematic validation of reference genes: a serious pitfall undervalued in reverse transcription-polymerase chain reaction (RT-PCR) analysis in plants. *Plant Biotechnol. J.* **6**, 609-618 (2008).
